# Cryogenic technology for infrared detection in space

**DOI:** 10.1038/s41598-022-06216-5

**Published:** 2022-02-11

**Authors:** Yinan Han, Ankuo Zhang

**Affiliations:** Department of Refrigeration and Cryogenic Engineering, Shanghai Ocean University, Shanghai, 201306 People’s Republic of China

**Keywords:** Energy harvesting, Aerospace engineering

## Abstract

Cryogenic technology has been developed cooperatively with infrared detection technology, which is of great significance for the development of space science and technology. To illustrate this coordinated development, the relationships between the infrared wavelength and the dark current in detectors with cryogenic temperature are analyzed, which shows the importance of cryogenic technology for infrared detection. Based on an analysis of infrared detection characteristics and cryogenic temperature requirements in different temperature zones, the development direction of cryogenic technology for infrared detection in space is analyzed and combined with the application of cryogenic technology in several key infrared space missions.

## Introduction

**Figure 1:**
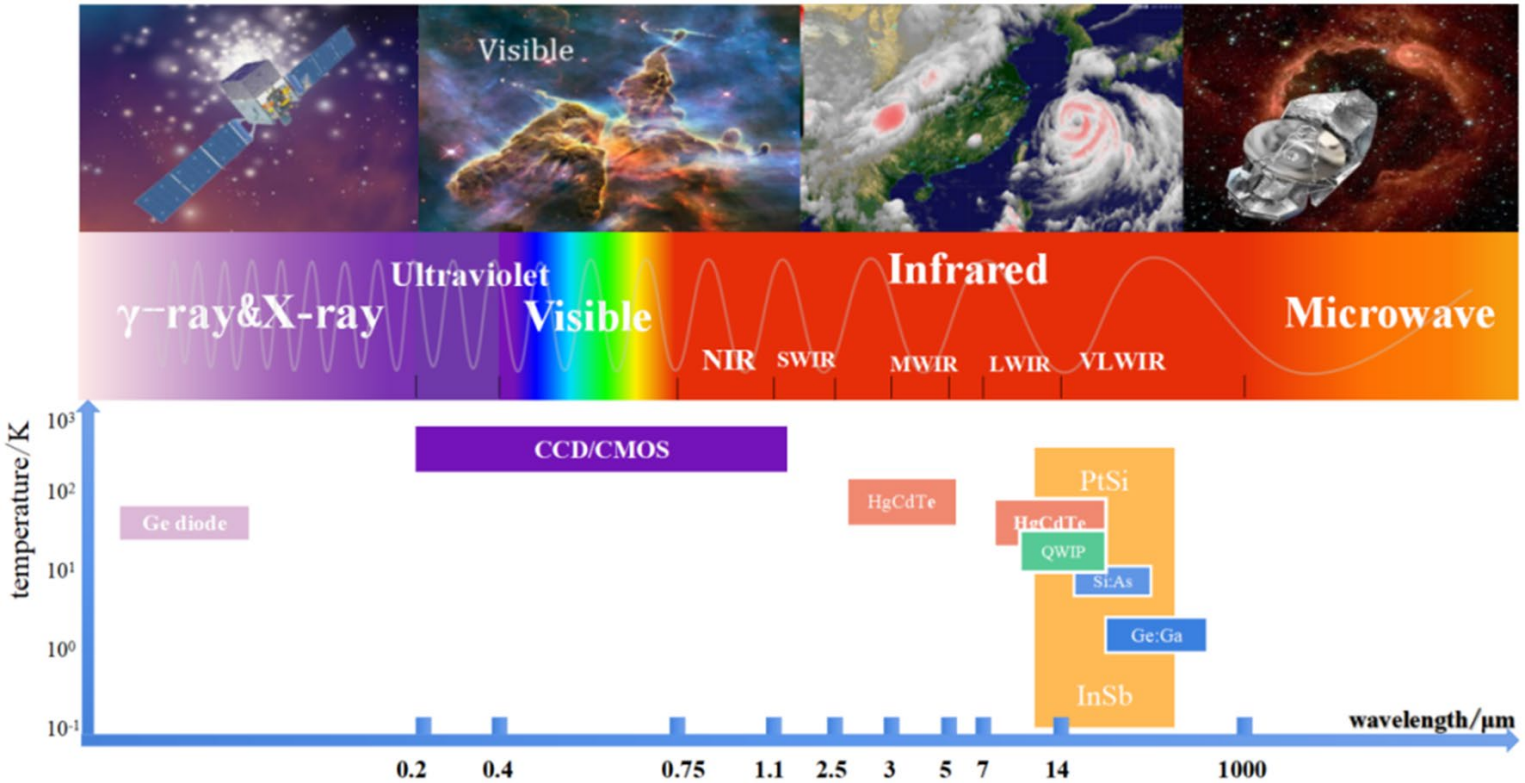
Working temperature maps of various detection wavelengths (generated by wps office, 11.1.0.11115-releaase, https://www.wps.cn/).

The broad spectra of optics includes microwaves, infrared, visible, and ultraviolet light, and X-ray and γ-rays, which are the main categories for astronomical observations, as shown in Fig. 1. Universal expansion causes the celestial emission wavelength to stretch constantly. Visible and ultraviolet light emitted by early stars has probably been extended into the infrared region, and thus infrared light has become an important branch of astronomical observation. However, most infrared radiation is absorbed by the Earth's atmosphere, which results in a certain limitation on ground-based astronomical observations. Therefore, the high sensitivity of infrared detection in space is particularly important^1^.

Existing detectors include photon detectors and thermal detectors. Photon detectors use incident photons to interact with bound electrons and are superior to thermal detectors in terms of sensitivity and response speed; thus, they are commonly used in space exploration missions^2^. Photon detectors are typically used in low-temperature environments because low temperature can effectively suppress dark current detection and background noise interference to improve detection performance. The operating temperatures of different types of cryogenic detectors are shown in Fig. 1.

Infrared detectors are core components for infrared detection technology, and cryogenic technology has been codeveloped with cryogenic infrared detectors. Infrared detector units began with small-scale first-generation multivariate detectors, developed with second-generation line and small-scale array detectors, and now widely use third-generation large-scale focal plane (256 × 256 ~ 4 K × 4 K) multicolor detectors^3^. The detection performance has been greatly improved; at the same time, the heat load of the device and heat radiation leakage has also increased, which requires a lower temperature and greater cooling power to guarantee the effectiveness of the detectors. Cryogenic space refrigerators with large cooling capacities (the cooling capacity classification is shown in Table 1 in the Supplementary Information) have been develop rapidly, such as China's Fengyun series satellites and the United States’ Landsat and GOES series satellites. Therefore, detector instrument replacement requires an increasingly stringent cooling capacity and temperature range, and the development of cryogenic technologies, especially for large cooling capacity and deep cryogenic technology, has been greatly promoted.

However, the space environment is challenging, and it is extremely difficult to achieve device maintenance while in orbit. Therefore, infrared detectors require high reliability and long-life components, especially cryogenic cryocoolers, whose performance determines the service life of infrared detectors. The development of long-life (> 5 years) and highly reliable space cryogenic coolers is synergistic and symbiotic with the development of modern infrared detection technology. For example, the Improved Stratospheric and Mesosphere Sounder (ISAMS) on UARS was successfully launched in 1991, which realized the first space application of Oxford Stirling refrigerators^4^; China’s Fengyun-4, Gaofen-5 and other satellites have also been launched for space application with long-life mechanical refrigerators^5,6^; their working lives have extended beyond the service lives of a low-temperature Dewar in the early Infrared Astronomical Satellite (IRAS) and Cosmic Background Explorer (COBE)^3^.

Based on the current development of cryogenic technology at different detection temperatures, this paper analyzes the influence of cryogenic technology on the infrared wavelengths and dark currents in detectors. The space coordination and symbiotic relationship of infrared detection technology and cryogenic technology is illuminated through the different temperature zones of the influence law of the detector. Finally, the development of cryogenic technology for future space exploration is summarized by comparing and analyzing the interdependent relationships between them in space projects.

## Cryogenic characteristics of infrared detector

**Figure 2:**
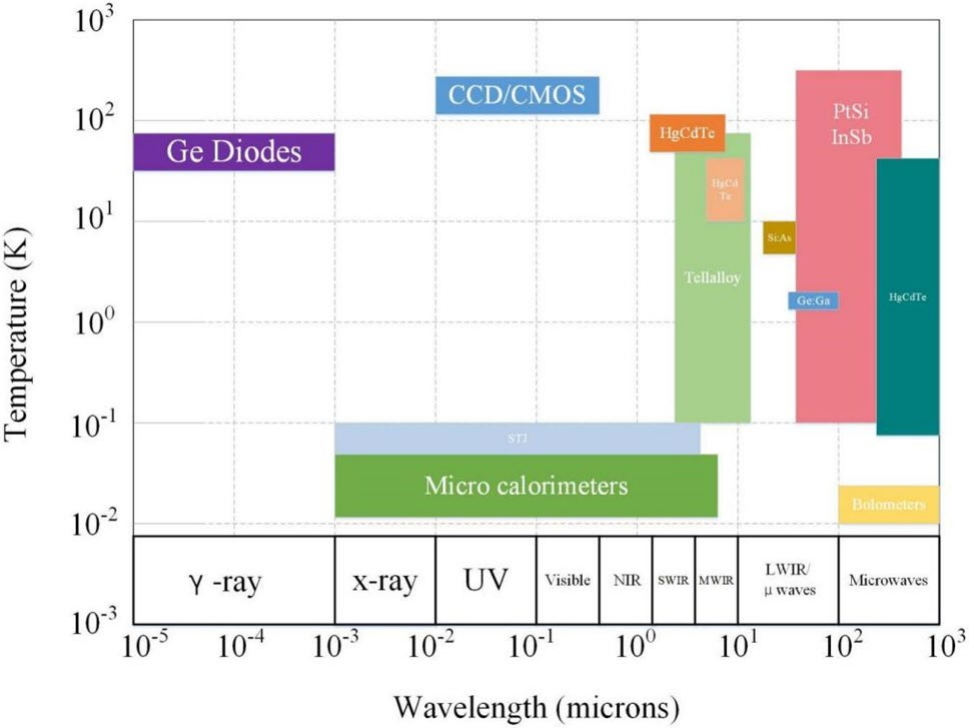
Common radiation detection types, wavelengths and operating temperatures^3^ (generated by Microsoft Visio 2013, 10.0.19043, https://www.microsoft.com/zh-cn/microsoft-365/visio/flowchart-software).

Several common types of radiation, infrared wavelengths and working temperatures of detectors are presented in Fig. 2. The operating temperatures of infrared detectors always focus on low temperatures between 2 and 170 K^3^. A higher operating temperature can cause thermal excitation, which results in noise interference, a reduction in detector performance and a sharp deterioration in the imaging quality of the target object. The above influences on detection can be minimized by providing an appropriate low-temperature environment. This section focuses on the relationships among low temperature, infrared wavelength and dark current in a device, and the necessity of cryogenic technology is illustrated for infrared detection.

### Relationship between infrared wavelength and low temperature

Wien’s displacement law is one of the basic laws of thermal radiation and is expressed as $\lambda T = b$, where b is Wien’s constant, λ is the detection wavelength, and T is the low temperature. The radiation wavelength shifts toward the shortwave direction as the temperature rises. In general, the longer the infrared wavelength is, the lower the operating temperature of the detector will be. Infrared detection wavelengths mainly consist of five categories: near infrared, shortwave infrared, medium wave infrared, longwave infrared (also called thermal infrared) and very longwave infrared. Under normal circumstances, a shortwave detector works at approximately 150 K, and a medium wave detector works at approximately 77 K. If the detector needs to work in the longwave or very longwave infrared spectrum, the operating temperature needs to be decreased to approximately 40 K or 20 K and below the temperature region^3^.

**Figure 3:**
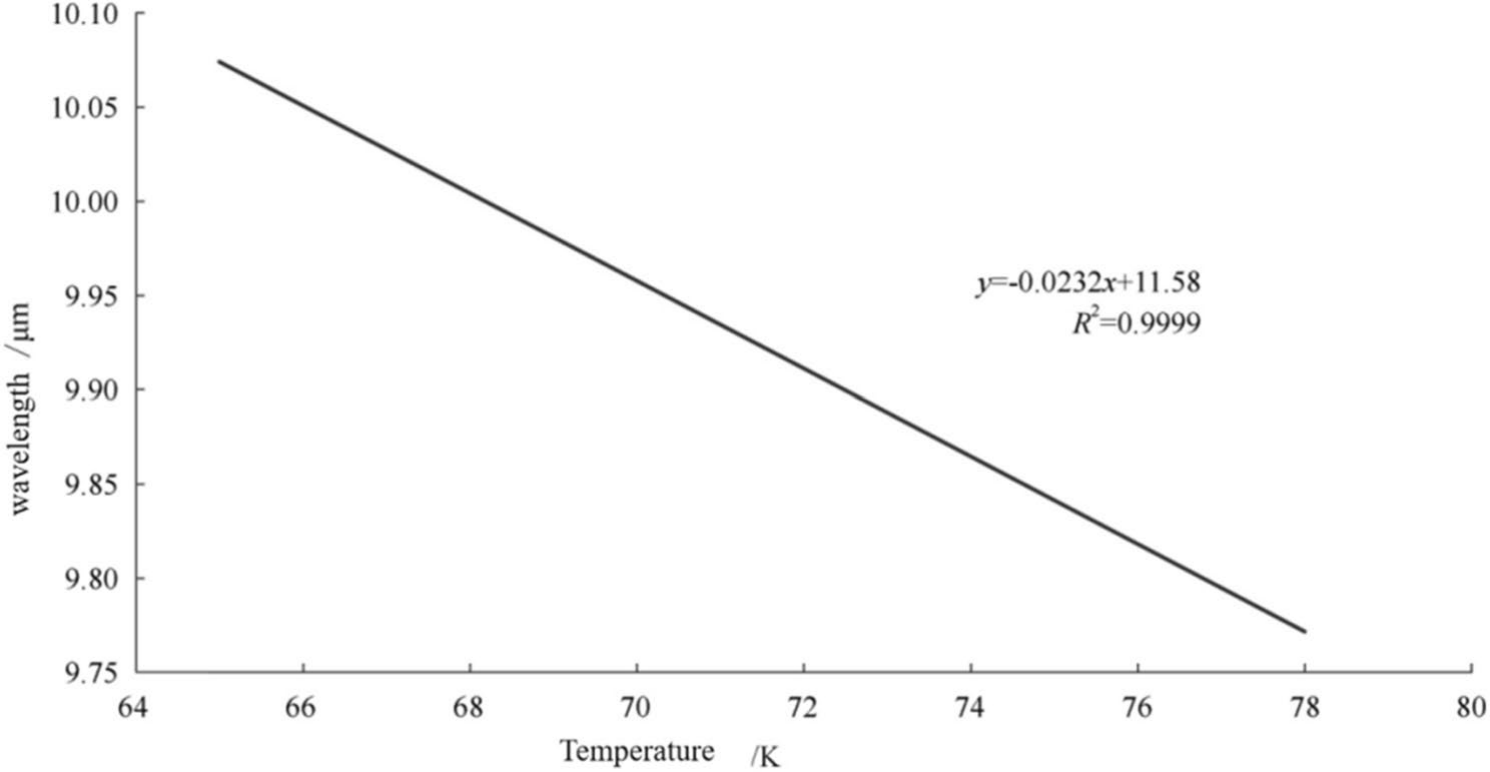
Relationship of wavelength and temperature of mercury cadmium telluride detector^7^.

Taking a mercury cadmium telluride (Hg_1−x_Cd_x_Te, MCT) detector as an example, Formula (1) and (2) show the relationships among the cutoff wavelength ($\lambda_{{\text{c}}}$), forbidden bandwidth ($E_{{\text{g}}}$) and temperature (T) if the premise of component × is determined. The forbidden bandwidth is proportional to the temperature, and the cutoff wavelength is inversely proportional to the forbidden bandwidth. It can be concluded that operation needs to be performed at a lower temperature when the wavelength is extended. The test results are shown in Fig. 3, and the wavelength of the longwave HgCdTe detector is extended by 0.0225 μm when the operating temperature is decreased by 1 K temperature^8^.

1 $$E_{g} = - 0.302 + 1.93x + 5.35 \times 10^{ - 4} \times T(1 - 2x) - 0.810x^{2} + 0.832x^{3} (eV)$$

2 $$\lambda_{c} = 1.24/E_{g}$$

### Relationship between low temperature and dark current in detectors

The noise interference formed by the dark current in a detector device has a significant impact on the detection, and low temperature is an effective way to suppress the dark current in the detector, which is proportional to the relationship between the two. The dark current increases as the operating temperature of the detector increases. For different detectors, their dark currents are of different magnitudes, and thus, the required operating temperature range also varies greatly. This subsection details the relationship between the operating temperature of the detector and the dark current, using several commonly used detectors as examples^8^.

**Figure 4:**
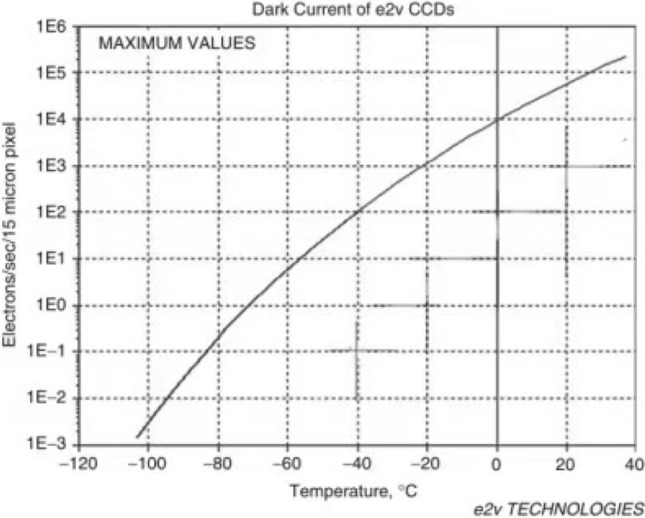
Relationship of dark current and operating temperature of CCD.

Figure 4^9^ shows a plot of dark current versus temperature for charge-coupled devices (CCDs), a semiconductor element developed in 1970 that has been widely used in remote sensing in space and other fields. In the absence of signal injection, dark currents are formed, but the number of dark currents is relatively small compared to other detectors, and the reading noise is low and causes less interference with the signal, so the operating temperature range is relatively high^10^. It can be seen in the figure that the operating temperature is approximately 170 K, where the number of dark currents is small and the interference to the detector is low.

**Figure 5:**
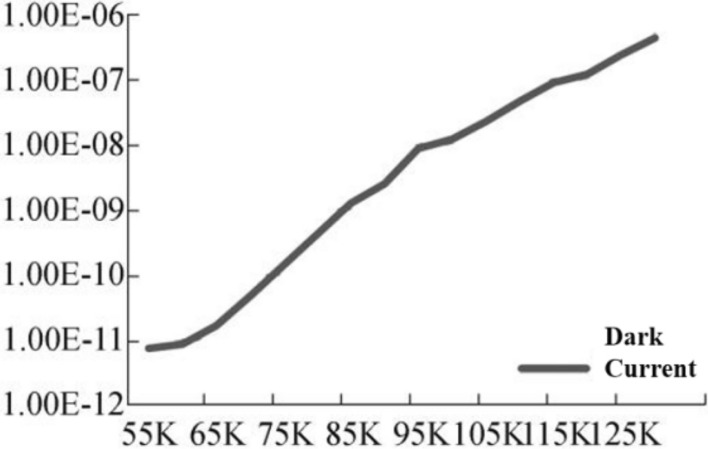
Relationship of dark current and operating temperature of HgCdT^7^.

Mercury cadmium telluride (HgCdTe) is a commonly used material in infrared detection and is a narrow-band semiconductor that can achieve efficient detection in a full spectrum by adjusting the component occupancy ratio^11^. Its technology is mature and has exhibited good performance, and the operating temperature is mostly concentrated around 77 K. Figure 5 shows the relationship between the dark current and the operating temperature of longwave HgCdTe detectors, and it can be seen that the operating temperature is linearly related to the dark current. When the temperature is greater than 65 K, the dark current varies drastically with temperature and has a great impact on detection, while when the temperature is less than 65 K, the dark current changes tend to stabilize.

**Figure 6:**
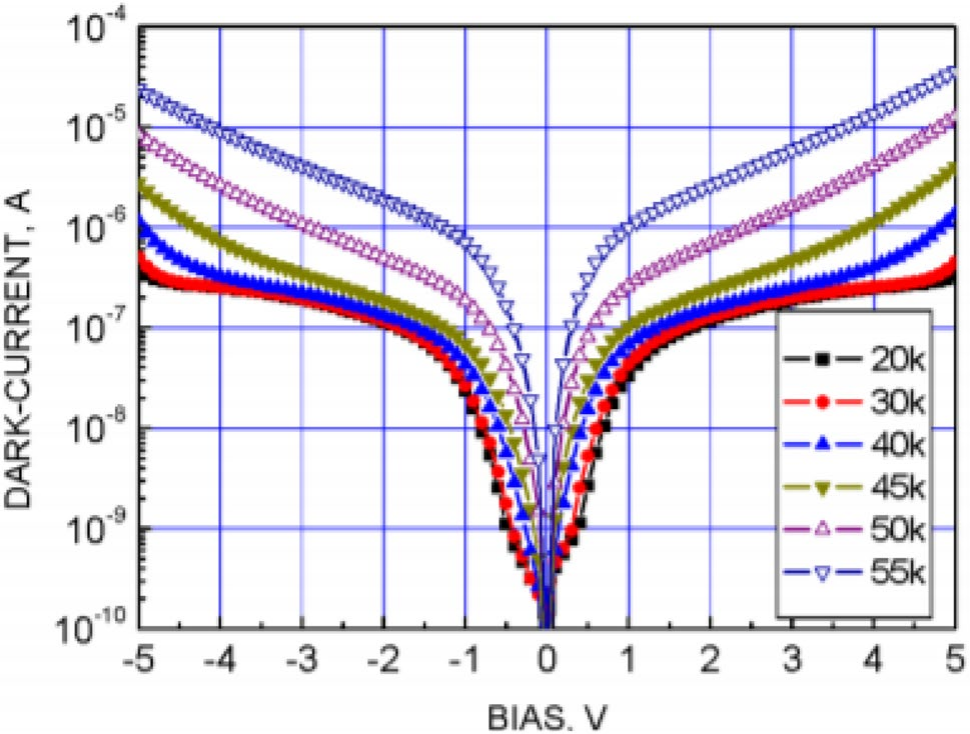
Relationship of dark current and operating temperature of QWIP^13^.

A quantum well infrared photodetector (QWIP) has the advantages of fast response speed and high resolution, and it is easy to prepare large arrays and multispectral imaging array detectors^12^. Compared with HgCdTe, a QWIP has more advantages in the detection of longwave and very longwave infrared signals, but it needs to work in a lower temperature zone. Figure 6 shows the relationship between the dark current and operating temperature of a very longwave QWIP, from which it can be seen that the variation in dark current with temperature tends to be stable when the working temperature reaches approximately 40 K, this temperature zone is beneficial for improving quantum efficiency and realizing efficient observation.

Type-II superlattice (T2SL) materials were applied in infrared detectors in 1987. These materials possess high homogeneity and low cost and are easier to prepare into large-scale homogeneous devices than HgCdTe. The material band gap is adjustable, and the spectral response can cover 2–30 μm. Their high quantum efficiency is more advantageous at LWIR and VLWIR spectra. In addition, a T2SL material design can suppress dark current, increase the focal plane operating temperature, and effectively reduce the cooling pressure^14^.

In 2007, Raytheon and JPL jointly reported a 256 × 256 longwave T2SL focal plane with an operating temperature of 78 K and a cutoff wavelength of 10.5 μm. In 2010, a 1024 × 1024 LWIR detectors with cutoff wavelengths of 11 μm were prepared. The background limited detectivity was 1.1 × 1011 at 87 K. Wuhan Guide IR prepared the first longwave surface array device with a size of 320 × 256 using T2SL IR detector material, a back cutoff wavelength of 10.5 μm, and a focal plane operating temperature ≥ 68 K^15^.

## Application of cryogenic technology for infrared detection in space

To explore the origin of the universe, scientists have launched many infrared observation satellites and optical telescopes in space. A large part of their detection systems contain infrared detectors. To achieve the high-quality detection of weak signals, cryogenic refrigerators have become a necessity for space missions. Cryogenic technology changes with the requirements of detectors in different operating temperature zones. This chapter describes the status and characteristics of the cooperative development of cryogenic technologies in the infrared detection in space for different cooling methods and temperature zones.

### Radiative refrigeration, mechanical refrigeration and high temperature zone detection

The wavelengths of detectors in high-temperature zones are mostly concentrated in the near-infrared and shortwave infrared spectra, and detectors with low dark current and high working temperature are typically used, such as CCD. Combined with the advantage of the cold environment of cosmic space, passive radiation refrigeration can be used. This has become the preferred cryogenic technology for early space detectors in high-temperature zones owing to its advantages of long operating time, low consumption and no vibration.

**Figure 7:**
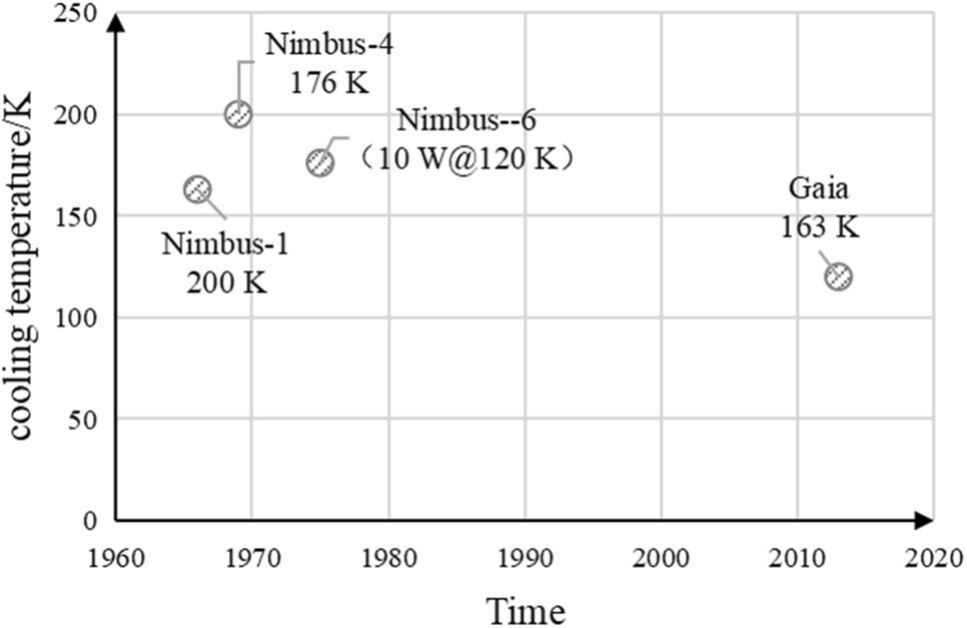
Radiative refrigeration performance for high temperature detection missions.

The first space application of radiation coolers was achieved on the U.S. Nimbus-1 satellite, which cooled the PbSe detector on the high-resolution infrared radiometer to 200 K^16^; the Kepler satellite launched by NASA in 2009 and the Gaia detector launched by the European Space Agency (ESA) in 2013 both used radiation cooling to cool the CCD focal plane^17^; and the Euclid satellite, expected to be launched in 2022, will orbit the solar-terrestrial L2 point with a good external thermal environment, and its visible and near-infrared focal planes will be cooled entirely by passive radiation cooling^18^. The radiative refrigeration parameters used for high operating temperature detectors are shown in Fig. 7.

However, radiation cooling can be easily affected by the operation orbit and placement, and the cooling capacity is small, which limits its application. At the same cooling temperature, the cooling capacity of mechanical cooling is much greater than that of radiation cooling, and it is not limited by the operation orbit, which is conducive to developing large-array infrared detectors.

**Figure 8:**
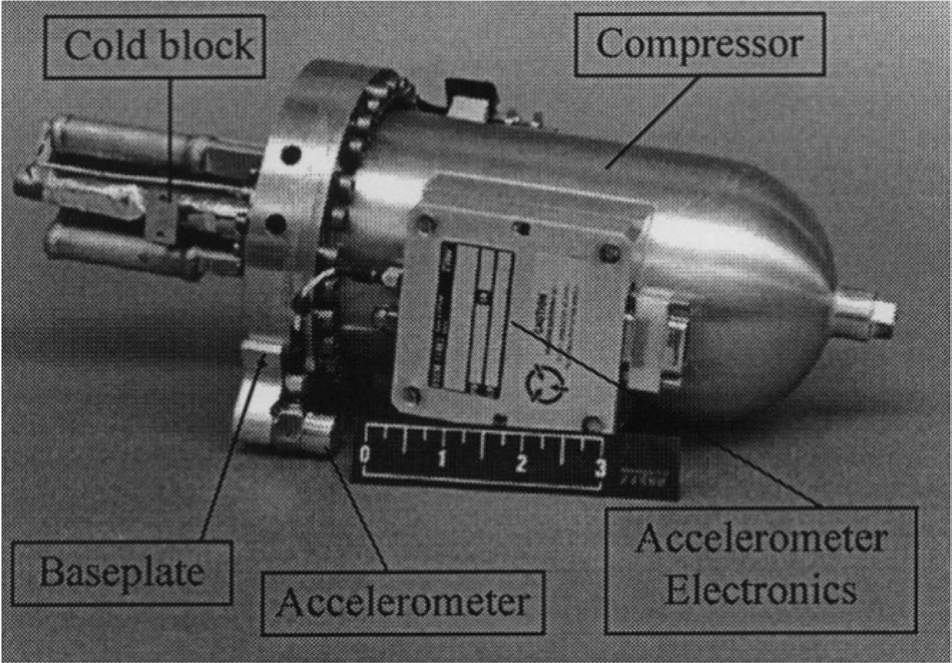
TRW miniature pulse tube cooler^20^.

**Figure 9:**
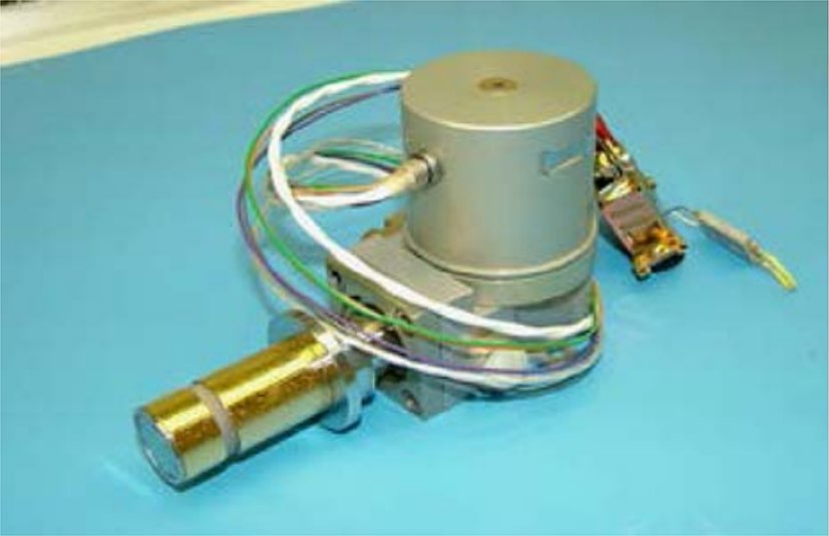
Ricor K508 miniature Stirling cryocooler^21^.

The miniature pulse tube cooler (Fig. 8)^19^ developed by TRW for U.S. military satellites in 1998 was a good start for pulse tube refrigerators in space exploration, cooling detectors to 150 K and weighing 2.25 kg. After continuous improvements, it was used for cooling the focal plane of the HSI and Hyperion spectral imagers, providing a cooling capacity of 1.63 W at 55 K. Its cooling efficiency is comparable to that of Stirling coolers; for the HOT infrared focal plane array, the Lockheed Martin Advanced Technology Center (LM-ATC) developed a coaxial miniature pulse tube refrigerator, which weighs only 328 g and has a cooling power of 690 mW at 150 K^20^; Israel Ricor has been devoted to the development of miniature cryocoolers for several years. The Ricor K508 (Fig. 9) is an integral rotary Stirling cooler that weighs only 450 g. The K508 was successfully applied in the Mars Science Laboratory (Curiosity) and cooled to 173 K^21^. The Ricor K527 is a micro split liner Stirling cooler and is used for IR imagers. The K527 weighs 270 g. Later, Ricor developed a short-finger model of the K527, and the cooling power reached 500 mW@150 K^22^. Germany AIM also reported several miniature Stirling coolers for HOT detectors, such as the 160 K SX020 cooler with a weight of only 240 g and the 140 K SX030 cooler with a weight of 380 g^23^.

**Figure 10:**
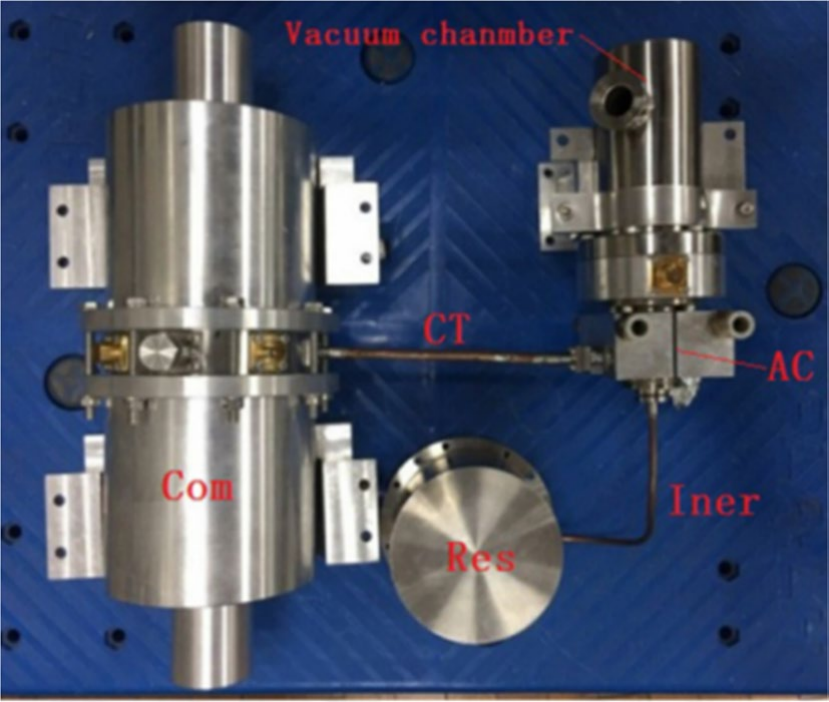
170 K pulse tube cryocooler^25^.

A 170 K pulse tube refrigerator (Fig. 10) was developed by the Shanghai Institute of Technical Physics of the Chinese Academy of Sciences (SITP) for space CCD cameras to improve their high-resolution detection capability. The pulse tube cooler is a coaxial type with a cold head length of approximately 66 mm and an overall weight of less than 12 kg, which can obtain a cooling capacity of 50 W at 170 K with an input power of less than 230 W and has a design life of more than 5 years^24^. China’s sky survey project will use a large cooling capacity pulse tube refrigerator to cool the focal plane, where the main focal plane consists of a CCD for imaging in the 0.25–1.05 μm range and requires an operating temperature below 188 K and a cooling capacity of 150 W, while the shortwave infrared focal plane requires a low temperature below 80 K and a cooling capacity of 6 W.

### Radiative refrigeration, mechanical refrigeration and 90 K temperature zone detection

The detection wavelength of the 90 K temperature zone is mostly concentrated in the near infrared to medium and far infrared regions. Cryogenic technology has been rapidly developed with the update of a series of satellite instruments, such as China's FengYun series of satellites (FY), the U.S. Geostationary Operational Environmental Satellite (GOES) and European Meteosat satellites.

All of the above mentioned satellites are representative meteorological satellites. The detection focal planes of the first two generations of GOES were cooled by radiation cooling until 2016, when the imager on the GOES-R satellite used a pulse tube cooler to cool the detector, making the spatial resolution significantly better than that of the previous satellites. The same is true for the Fengyun satellites: FY-1, 2 and 3 all used radiation cooling until the atmosphere vertical sounder (GIIRS) in FY-4 used a Stirling cooler to cool the detector, making it possible to achieve high precision detection in the vertical direction. Meteosat Third Generation (MTG) comprises the Imagine satellite (MTG-I) and the sounding satellite (MTG-S). Similarly, MTG replaced the passive refrigeration used on previous Meteosat satellites with an active mechanical cryocooler, and continued to increase capabilities in climate detection^25^.

**Figure 11:**
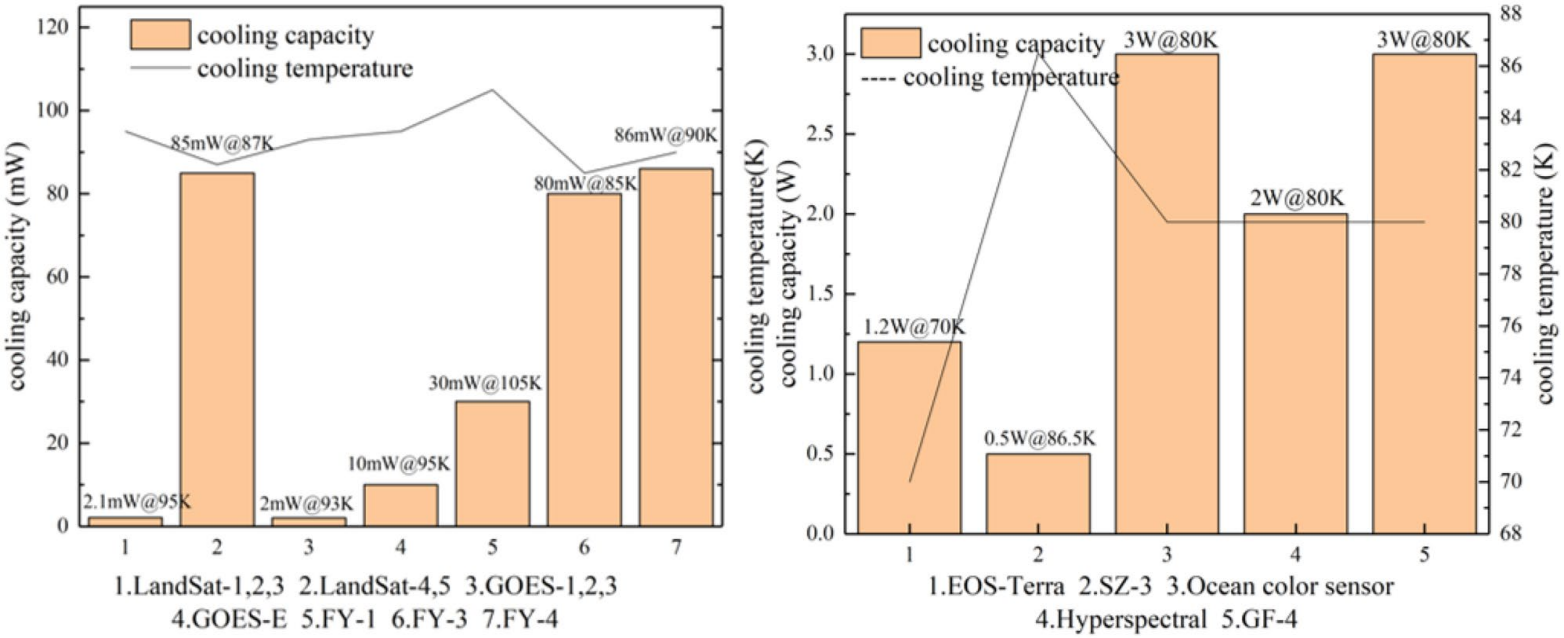
Refrigeration performance parameters in the 90 K detection temperature zone (the left is radiative cooling, the right is mechanical cooling) (generated by originpro 2017, b9.4.2.380, https://www.originlab.com/).

There are many other applications of mechanical cooling technology in the detection temperature zone, such as the Advanced Space-borne Thermal Emission and Reflection Radiometer (ASTER) instrument in the U.S. EOS system, which detects shortwave infrared and thermal infrared signals and uses two Stirling coolers to cool detectors to an operating temperature of 80 K, with an on-orbit performance of 1.2 W@70 K^26^; the high-resolution imaging spectrometer on China’s ShenZhou-3 (SZ-3, 2002) satellite used a dual-drive Stirling refrigerator developed by SITP to cool the infrared focal plane to 85 K, which was the first space application of domestic mechanical refrigerators^27^. The pulse tube refrigerator developed by the Technical Institute of Physics and Chemistry (CAS) was used to cool the mid-wave infrared focal plane on the GaoFen-4 satellite (GF-4, 2016). With a cooling capacity of 3 W@80 K and a design life of more than 10 years, it was the first space application of long-life pulse tube refrigerators in China^6^. Tang et al.^28^ carried out research on micro (1 kg) pulse tube refrigerators in space, and the cooling capacity of the microspace pulse tube cooler can reach 1.2 W/35 W in the 80 K temperature region. With 45 W input power, the cooling capacity per kilogram reaches 1.54 W. SITP has developed two miniature 80 K pulse tube refrigerators for cooling the space ocean color sensor and hyperspectral imager: one weighs 4.5 kg with a design life of 5 years and can obtain a cooling capacity greater than 3 W with an input power of 74 W, while the other has a mass less than 1.5 kg, and its cooling capacity can exceed 2 W when the input power is more than 55 W^24^. The cooling performance parameters of each task are shown in Fig. 11.

### Mechanical refrigeration and 60 K temperature zone detection

The detection wavelength in the 60 K temperature zone is mostly concentrated in the thermal infrared and has applications in earth remote sensing resource satellites and space telescopes, such as China's Fengyun-4, Gaofen-5, Hubble Space Telescope (HST), NASA Earth Observation System (EOS), GOES-R and MTG satellites. For more advanced detectors, long-life continuous cooling is also very important while ensuring cooling efficiency.

The Near Infrared Camera and Multi-object Spectrometer (NICMOS) on the Hubble Space Telescope (HST), launched in 1990, initially adopted a solid N_2_ Dewar to cool the detectors to 58 ± 2 K. However, greater than expected heat leakage caused the cooling mission to remain for only 23 months and well below the design lifetime of 5 years. After five years, the cooling was taken over by a turbo-Breton refrigerator, allowing the instrument to operate again^29^. This mission exposed the shortcomings of cryogenic Dewar technology, which severely affects the operating lifetime of detectors due to the amount of refrigerant carried and unavoidable thermal leakage.

**Figure 12:**
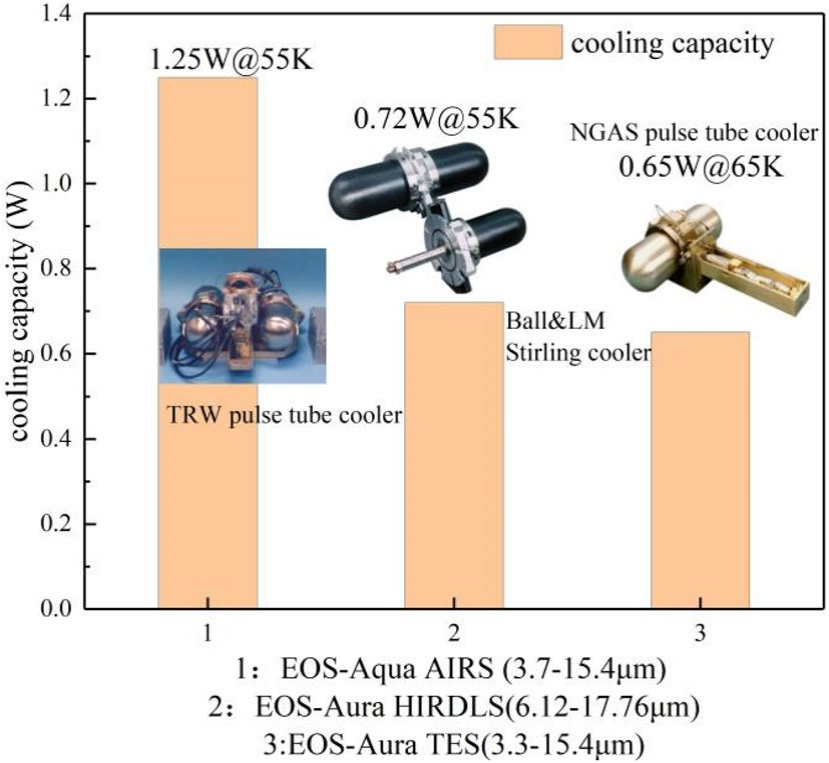
Refrigeration performance of EOS satellites (generated by originpro 2017, b9.4.2.380, https://www.originlab.com/).

After the introduction of the Oxford-Stirling cryocooler, the NASA EOS series of space science instruments began to adopt mechanical refrigeration to cool the IR detector. Figure 12^30–32^ shows the performance parameters of some models. The early Oxford cooler applied the split structure design of a single piston compressor and a single expander. The vibration force is close to 50 N during work, which is unacceptable for space applications (generally 0.2 N). The BAe Stirling cryocooler used in EOS/MOPITT (Measurements of Pollution In The Troposphere) adopts the Lockheed digital error feedback control method to make the axial vibration less than 0.2 N. The Fujisu Stirling cryocooler used in EOS/ASTER also adopts active vibration suppression, which can reduce the axial vibration force to 0.1 N in a frequency range of 40–135 Hz^33^.

**Figure 13:**
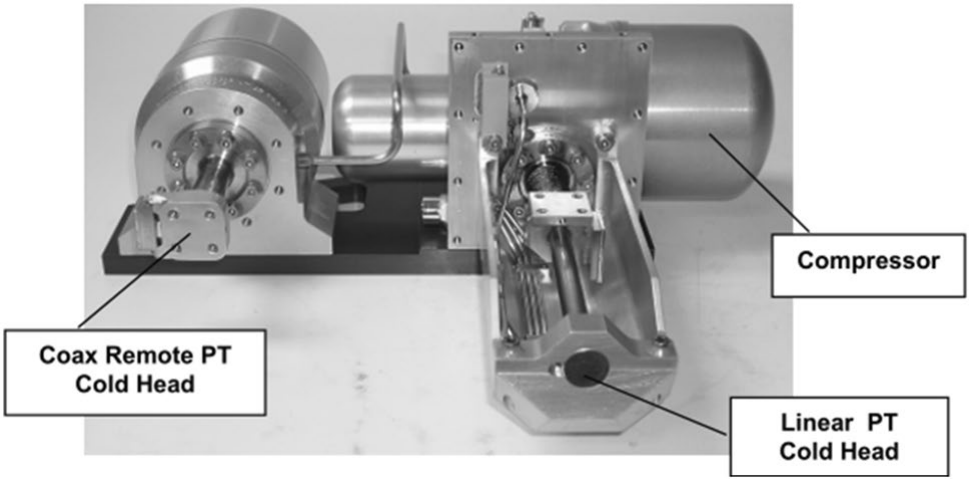
ABI pulse tube cooler^34^.

GOES-R is NASA’s new generation of weather satellites, it has colder detectors on the focal plane of the advanced baseline imager (ABI) and its image resolution has been greatly improved. Detectors were cooled by the NGAS's two-stage pulse tube refrigerator with a long life (design life of 10 years), low quality and efficiency, which integrates linear and remote coaxial cold fingers (Fig. 13) to provide cooling power at both temperatures simultaneously and obtain a cooling capacity of 1.9–2.3 W at 53 K and 5.1–8.0 W at 183 K^34^.

**Figure 14:**
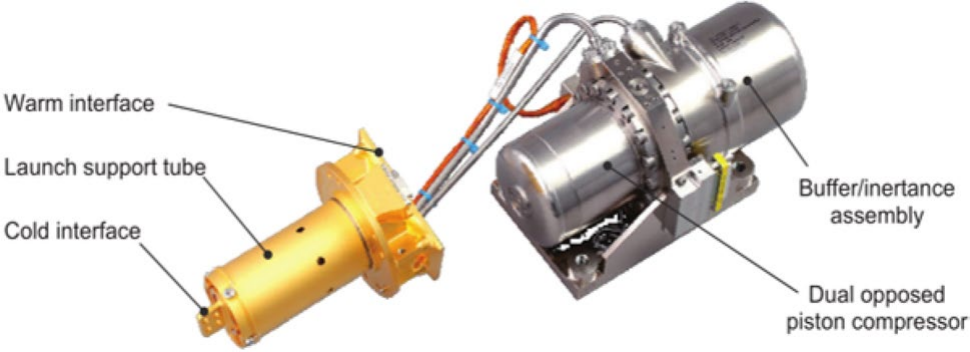
Air liquid pulse tube coolers (LPTC)^35^.

MTG is the third generation Meteosat satellite, which adopts large pulse tube coolers (LPTC, Fig. 14) manufactured by Franch Air Liquid company to cool the infrared sounder (IRS) and flexible combined imager (FCI) on the MTG. The focal planes are cooled to 50–55 K. The LPTC cold finger adopts a coaxial structure, which is convenient for coupling with the focal planes. The LPTC can provide 2.3 W@50 K with a cooling power of 160 W^35^. Another miniature pulse tube cooler (MPTC) produced by Air Liquid was used in the ESA Sentinel-3 mission, and its weight was 2.8 kg. The MPTC can meet the requirements for flight projects of 15 to 20 years. Both the LPTC and MPTC adopt dual opposed piston flexure bearing compression to counteract the axial vibration force. By integrating passive isolation dampers and low-frequency drive electronics with an active vibration suppression system, it is possible to achieve a lower vibration level^36^.

**Figure 15:**
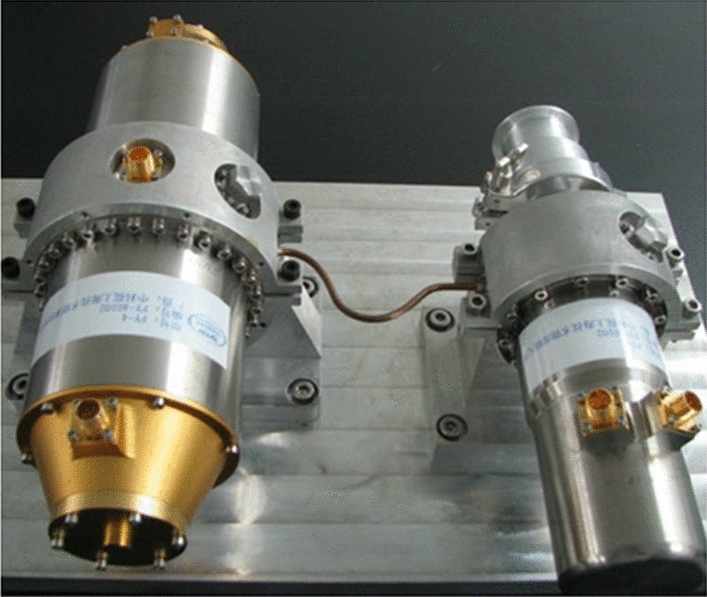
FY-4 Stirling cryocooler^37^.

The GIIRS on China’s FY-4 adopts the long-life Stirling refrigerator (Fig. 15) developed by SITP to cool a medium-longwave infrared detector with a cooling capacity of 2.3 W@60 K and a design life of more than 7 years^37^. To meet the cryogenic requirements of next-generation high-resolution infrared multispectral cameras and spectral imagers, SITP also developed a 60 K single-stage pulse tube cryocooler, which weighs 8.5 kg and effectively provides more than 6 W of net cooling power with an input power of 195 W^38^. The visual and infrared multispectral sensor (VIMI) is one of the payloads of Gaofen-5 (GF-5, 2018), and the cooling system uses an 83 K pulse tube cooler to cool the shortwave and medium-wave infrared detectors and a 60 K pulse tube cooler to cool the longwave detectors, which uses an opposed piston compressor to effectively reduce the vibration interference to the detectors^39^.

### Miniaturized refrigerator and low temperature zone detection (< 60 K)

The low-temperature zone is mostly used for a large focal plane array and multispectral detection: detection wavelengths extend to the very longwave infrared, and the working temperature of detectors is generally approximately 40 K. A multistage refrigerator can meet the cryogenic requirements of this detection zone. Because of its volume and more complex structure, it can easy burden the whole machine and increase instability. Most single-stage refrigerators have a simple structure, which is conducive to the reliability of detection. Therefore, miniaturization and lightweight cryocoolers are important elements for increasing the reliability and operating time of detection missions and are also important research topics in space coolers.

The simultaneous detection of multiple spectral segments was realized (8.8–14.6 μm, 4.4–6.0 μm) in a geosynchronous imaging Fourier transform spectrometer (GIFTS) using the two-stage pulse tube cooler developed by Lockheed Martin Space Systems Company to cool the focal plane to 55 K. The refrigerator weighs 8.8 kg and provides a cooling capacity of 1.5 W at 55 K with 180 W of power input^40^.

The focal plane of the QWIP in Landsat-8 detects a thermal infrared zone of 8–12 μm and is cooled by a two-stage Stirling refrigerator developed by Ball Aerospace Technologies company, with a cooling capacity of 2 W@38 K, which is still operating normally^41^.

**Figure 16:**
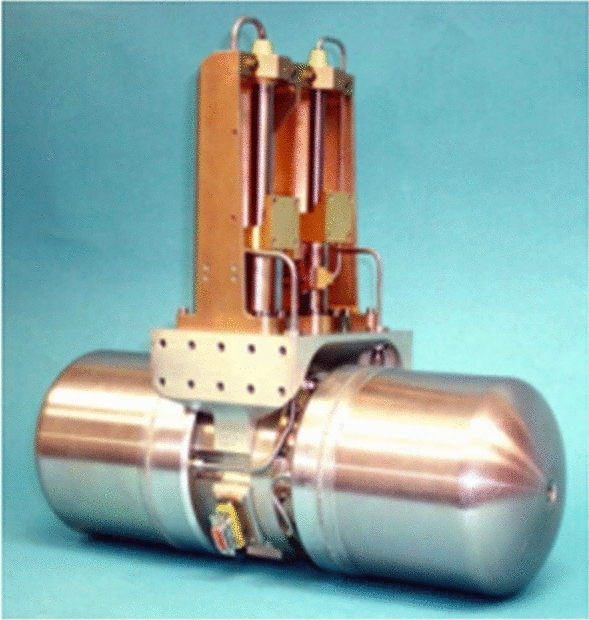
NGAS two-stage high capacity cryocooler^42^.

In 2003, NGAS reported a high-power two-stage pulse tube refrigerator (HCC) (Fig. 16)^42^, with a total weight of 14.3 kg for cooling the longwave infrared focal plane and optical devices on the SBIRS-Low satellite for a space-based infrared system. When the input power is 500 W, HCC can obtain a large cooling capacity of 17.5 W@85 K and 2.16 W@35 K with little interference and stable refrigeration performance. However, HCC is targeted for large cooling capacity with larger overall volume and mass, which is not suitable for application to small cooling detection. Therefore, NGAS followed up with small high-frequency coolers, such as a 35 K single-stage high efficiency cooler (HEC), which uses a coaxial pulse tube cold head for cooling IR focal planes in scientific satellites, and the development of a micropulse tube cooler (MCP), which provides long-life cooling for hyperspectral and infrared imaging in tactical airborne and space applications. When the ambient temperature is 300 K, the efficiency can reach 1.5 W@46 K with 150 W input power^43^. Reliability and ten-year lifetime are among the features of these pulse tube coolers.

A 40 K pulse tube refrigerator was developed by SITP for longwave QWIPs, weighing 11 kg and providing 3 W of cooling power when the input power is less than 225 W^24^.

### Comprehensive refrigeration and liquid helium temperature zone detection

A large portion of deep space exploration, such as space telescopes, infrared astronomical satellites and balloon-borne space missions, operates near the liquid helium temperature zone, even in the mK temperature zone. The main method of refrigeration in deep space detection is comprehensive refrigeration technology, which ensures the stability and long life of operation while achieving performance. For sub-Kelvin cooling (below 300 mK), the commonly used ultralow temperature technologies are adiabatic demagnetization (ADR), ^3^He sorption and dilution refrigeration^44^.

The Spitzer Space Telescope (SST,2003), which was officially retired in January 2020, has been in orbit for more than 16 years, with a full payload uptime of 5.5 years, and has made significant discoveries in the field of space detection. Benefitting from the experience of a liquid helium Dewar in IRAS, the SST used a combination of radiation refrigeration and a liquid helium refrigeration solution. The satellite shell is cooled to 34 K through radiative heat exchange with the external environment. Then the thermal coupling between the telescope and shell was cut off, and the cryogenic gas could cool the optical system to 5.5 K and the focal plane detectors to 1.4 K. The evaporation of helium is reduced by radiation cooling, thus reducing the amount of liquid helium refrigerant carried and using only 360 L liquid helium to the requirement of a 5-year operating life^18^, compared to 720 L of liquid helium used for 10 months in IRAS and 600 L of superfluid helium used for only 305 days in COBE.

The Japanese infrared astronomical satellite ASTRO-F observation equipment contains an infrared camera (IRC) and a far infrared survey (FIS) operating at the 2–26 μm and 50–180 μm^45^, respectively. The cooling system consists of a two-stage Stirling cryocooler and a liquid helium Dewar to cool the focal planes. The two-stage Stirling cryocooler is a split type with an opposed piston structure, which allows the infrared detectors to be kept away from the vibration interference during detection with a cooling capacity of 0.2 W at 20 K with 90 W of power consumption^46^. The presence of a two-stage Stirling refrigerator not only reduces helium evaporation and extends the service life of liquid helium but also continues to provide cooling to ensure the normal operation of near-infrared detection after the liquid helium is fully depleted, thus extending the detection lifetime.

Sub-Kelvin cooling technology is important for infrared detectors in space and has been applied in Herschel, infrared telescope in space (IRTS) and Planck missions. The Herschel cooling system adopts a ^3^He sorption cooler manufactured by RAL to cool the focal plane with a cooling of 10 μW at 300 mK. Using activated carbon or molecular sieves to adsorb and decompress ^3^He can obtain a cooling temperature below 300 mK. This is the key technology of sub-Kelvin coolers. The Herschel optical system refrigeration relies on a sunshield to block sunlight and utilizes cold space to make it work at 80 K. The sub-K cooling design of the IRTS is similar to that of Herschel. However, space-borne sorption coolers include a sorption pump, and the impact of the pump vibration on the detector should also be considered^47,48^.

The Planck satellite (Planck, 2009) launched by ESA to detect the cosmic microwave background and achieved an mK-level cooling temperature zone for the first time using an active refrigeration system with nonliquid helium cooling. The refrigeration system consisted of a 20 K hydrogen adsorption refrigerator, a 4 K Joule–Thomson (J–T) refrigerator and a 100 mK helium dilution refrigerator. The sorption cooler transmits 1 W of cooling capacity at 18–20 K for the low-frequency device and provides precooling for the J–T cooler. Finally, the J–T cooler cool dilution refrigerator was used for a high-frequency instrument. The cooling system has an operating lifetime of 1 year^49^, which is the first successful application of J–T throttle refrigeration for space exploration missions in liquid helium temperature zones, as well as the first space mission of dilution refrigerators, marking the rapid development of sub-K cryogenic technology in the field of infrared detection.

The Experiment for Cryogenic Large-Aperture Intensity Mapping (EXCLAIM,2019) is a balloon-borne far infrared telescope. The combination of a cryogenic telescope and a medium-resolution (512) spectrometer allows the telescope to observe effectively in a dark region at lower altitudes. The telescope and spectrometers were placed in a Dewar with 2500 L of liquid helium. The Dewar provides 24 h of 1.7 K operation at a float altitude. ADR provides approximately 1 μW of cooling power for the spectrometer focal plane at 100 mK. The intermediate stage of 900 mK is cooled by a ^4^He adsorption cooler. Its design is similar to the Absolute Radiometer for Cosmology, Astrophysics, and Diffuse Emission II (ARCADE II) and the Primordial Inflation Polarization ExploreR (PIPER) instruments^50^.

**Figure 17:**
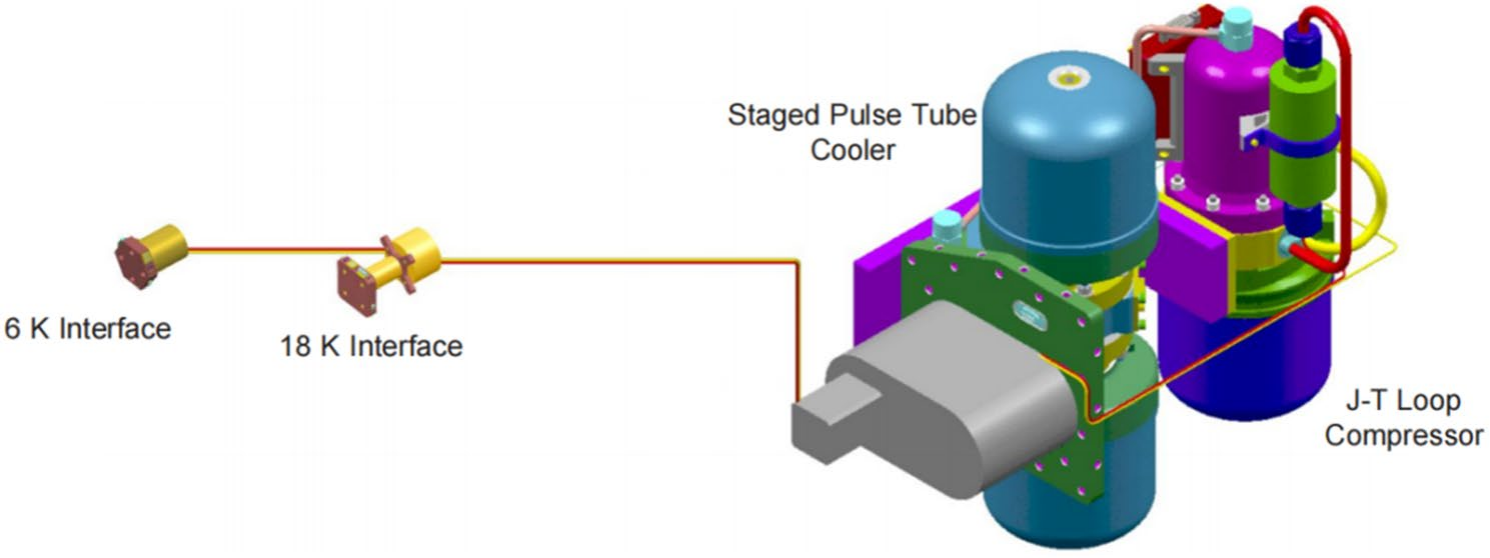
JWST MIRI cooler^52^.

The James Webb Space Telescope (JWST), expected to launch in 2021, is the first space mission to replace Dewar cooling with mechanical refrigeration and provides a source for future mechanical refrigeration applications in the deep cryogenic zone. The telescope optical system can be passively cooled to 30–50 K by a 5-layer sun visor without additional cooling^51^. The telescope carries the mid-infrared instrument (MIRI) with a three-stage pulse tube refrigerator coupled to a J–T refrigerator (Fig. 17). This scheme was proposed by Northrop Grumman Aerospace Technologies (NGST), which first precooled the J–T refrigerator to 18 K by a three-stage pulse tube refrigerator, and then cooled the detector by the J–T refrigerator. The cooler can obtain a cooling capacity of 113 mW with an input work of 395 W at 6.2 K^52,53^. The design of remote cooling solves the heat dissipation problem of the cooler and allows the detector to be away from the vibration and interference of the compressor, improving the stability of the entire detection process.

## Development characteristics of cryogenic technology for infrared detection in space

Cryogenic technology has developed cooperatively with infrared detection technology, from small unit detection development to today’s multicolor, multiband, large plane array and deep space detection. Different space detection requirements can promote the rapid development of cryogenic technology, such as large focal plane arrays with high thermal loads to promote the development of cryogenic technology with large cooling capacity; long-life infrared detection needs to promote the development of long life and high reliability cryogenic technology; and very-long wavelength detection missions with high sensitivity to promote the development of comprehensive refrigeration technology.

**Figure 18:**
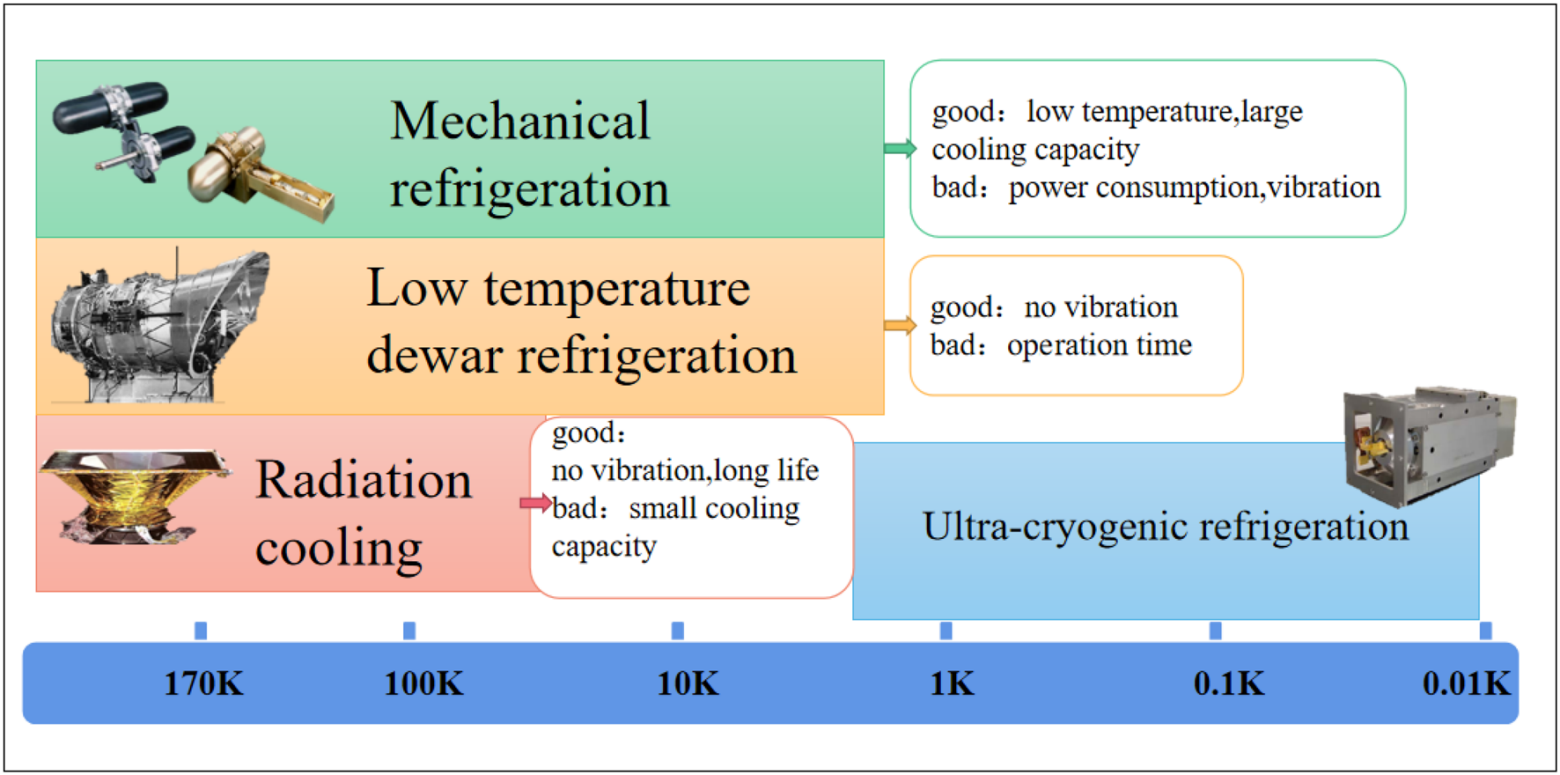
The main cryogenic technologies for space detection and its characteristics (generated by wps office 11.1.0.11115-releaase https://www.wps.cn/).

**Figure 19:**
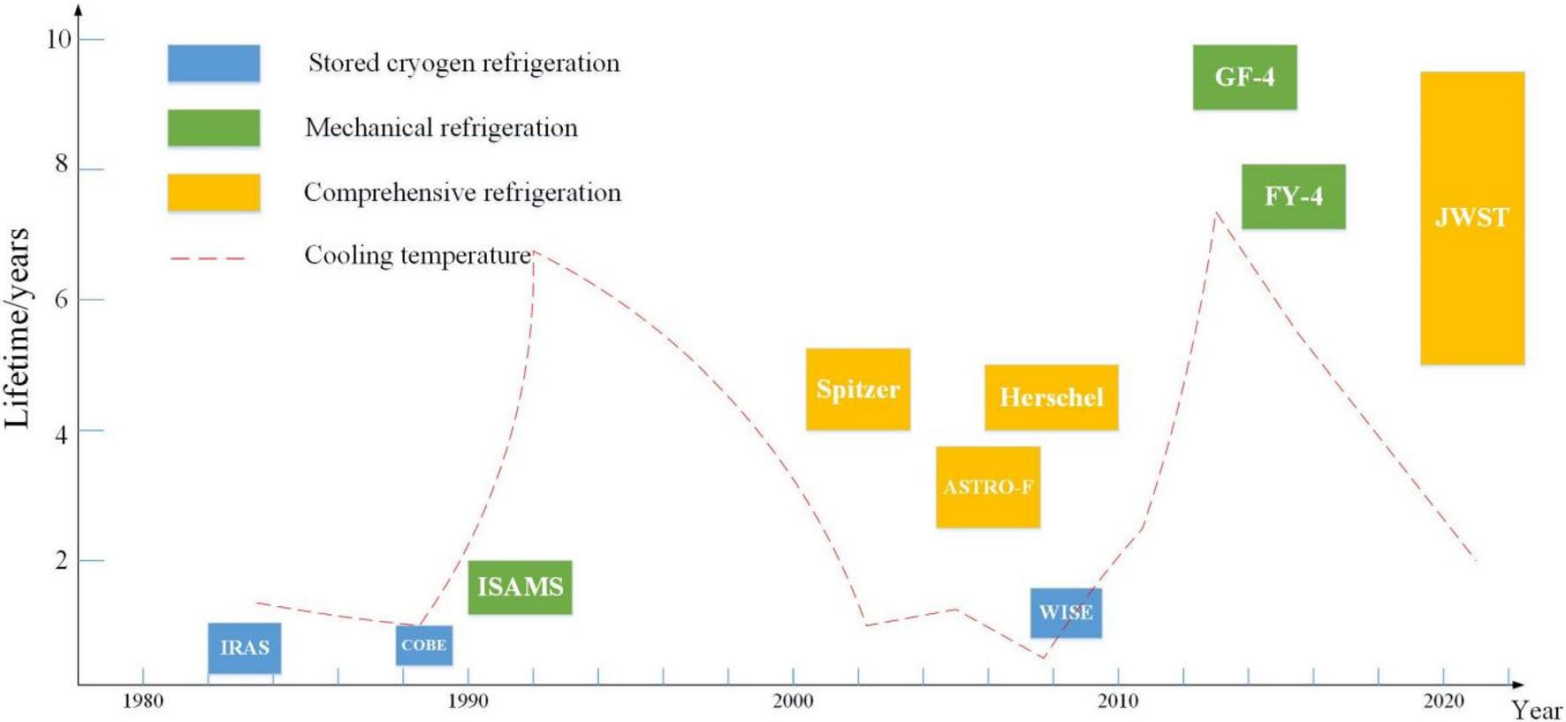
The main cryogenic technologies for space detection and its characteristics^54^ (generated by Microsoft Visio 2013 10.0.19043 https://www.microsoft.com/zh-cn/microsoft-365/visio/flowchart-software).

According to the applications of cryogenic technology for space infrared detection, cryogenic technologies mainly include mechanical refrigeration, radiation refrigeration, cryogenic Dewar refrigeration and ultra-cryogenic refrigeration (as shown in Fig. 18), which have made great progress in recent decades in terms of cooling temperature, cooling efficiency, operating life and reliability, as shown in Fig. 19.

In summary, cryogenic technology development for space infrared detection has the following characteristics:

1. Passive refrigerators are being replaced by active refrigerators. Due to high cooling temperature zones, small cooling capacity, space environment, orbit and location factors, the applications of passive radiation refrigerators are limited. Passive refrigeration has been unable to meet the low temperature requirements of many thermal infrared and very long wave infrared detectors. Active refrigeration can reach a lower temperature than passive cooling and provide better performance for IR detectors, which helps the sustainable development of large array detector technology in space.
2. Single refrigerations are being replaced by comprehensive refrigerations. Comprehensive refrigeration technology is being developed for deep temperature detection in space. The cooperation of various refrigeration technologies not only benefits to achieving a lower cooling temperature but also solves the heat dissipation problem of the refrigerators, thus maintaining a better cryogenic environment and providing efficient and stable long-term refrigeration for infrared detection.
3. In terms of miniaturization and light weight. Even though stored cryogen cooling has been widely used in early IR missions, its large volume and heavy mass can deteriorate the mission duration. The trend in space cooling in recent years is to develop a free-cryogen cooler, which is reflected in the Planck and JWST missions. In addition, HOT detector technology increases the operation temperature of the focal plane to the HOT range and is beneficial to improve cryocooler efficiency. It is possible to reduce the SWaP (size, weight and power consumption) of the cooler and promote the development of micro cryocoolers, such as the Lockheed Martin, Ricor and AIM, which have successfully developed many microcoolers for space. Miniaturization is also beneficial to reduce the vibration and cost of the cryocooler and increase the lifespan of the cryocooler.
4. In terms of microvibration, vibration generated from mechanical cryocoolers is a key element for space applications and will cause focal plane imaging to be blurred and deteriorate the resolution and accuracy of the detection target. In addition, vibration can easily cause the mechanical resonance of other instruments in satellites and interfere with sensitive sensors. The measures of vibration suppression mainly include the dual piston compressor structure and the addition of dampers at the displacers, which are applied in MTG and EOS.
5. The cooling temperature of the optical system has increased from a few K (such as ASTRO-F and Spitzer) to tens of K (such as Herschel and JWST). The refrigeration method has also changed from stored cryogen cooling to sun visors and space cold environment refrigeration. The effective cooling of the optical system can reduce the radiative loading on the detectors and provide a better working environment for detection.

## Conclusion

Cryogenic technology has codeveloped with space infrared detection technology, and low temperature can effectively suppress the detection of dark currents and background noise. The low-temperature influences on the detection wavelength and dark current have been analyzed, and the relationship between the radiation wavelength and temperature have been illustrated through the Wien displacement law, which shows an inversely proportional relationship between the working temperature and detection wavelength. The internal dark current characteristics of detectors at different operating temperatures have also been analyzed. Combined with cryogenic technology applications in space missions, the development characteristics of cryogenic technology for infrared detection in space have been analyzed by the detection characteristics and cryogenic requirements under different temperatures, and it can be seen that cryogenic technology changes greatly in cooling methods. Passive refrigerators are being replaced by active refrigerators because of the low temperature requirements of many thermal infrared and very longwave infrared detectors. Single refrigerators are being replaced by comprehensive refrigeration because of deep temperature detection requirements in space. In addition, the cooling efficiency, service time and reliability have been greatly improved with the development of infrared detection in space with large arrays, multispectral segments and high sensitivity. Cryogenic technologies are introducing lower temperatures, larger cooling capacities, longer life and miniaturization, which are more adapted to the needs of future infrared detection in space.

## Supplementary Information

Supplementary Information.

## Abbreviations

UV: Ultraviolet
NIR: Near infrared
SWIR: Short-wave infrared
MWIR: Medium wave infrared
LMIR: Long wave infrared
VLMIR: Very long wave infrared
*λ*: Wavelength (μm)
CCD: Charge-coupled device
CMOS: Complementary metal–oxide–semiconductor transistor
HgCdTe: Mercury cadmium telluride
QWIP: Quantum well infrared photodetector
*T*: Temperature (K)
*λ*_c_: Cutoff wavelength (μm)
