# Supplementary material for: Cryogenic technology for infrared detection in space
Source: Sci Rep. 2022 Feb 11;12:2349. doi: 10.1038/s41598-022-06216-5 (PMC8837754; doi:10.1038/s41598-022-06216-5)
Supplement: Supplementary file 1 — Supplementary Information. [file 41598_2022_6216_MOESM1_ESM.docx]

APPENDICES

**Cryogenic technology for infrared detection in space**

Yinan Han, Ankuo Zhang*

*Department of Refrigeration and Cryogenic Engineering, Shanghai Ocean University, Shanghai 201306, PR China. *email:*zhangankuo@126.com

*Table A1. Refrigerator classification by refrigeration temperature and refrigeration capacity [1]*

| category | Cooling capacity | | | | |
| --- | --- | --- | --- | --- | --- |
|  | 1 K | 4 K | 20 K | 80 K | 120 K |
| microminiature | - | ＜50 mW | ＜0.20 W | ＜0.8 W | ＜1.5 W |
| miniature | - | ＜0.2 W | ＜2 W | ＜6 W | ＜10 W |
| small | ＜0.3 W | ＜5 W | ＜30 W | ＜600 W | ＜1.0 kW |
| middle | ＜25 W | ＜100 W | ＜1 kW | ＜15 kW | ＜25 kW |
| large | ＞25 W | ＞100 W | ＞1 kW | ＞15 kW | ＞25 kW |

**References**

1. Chen, G. B., & Tang, K. Principle of small cryogenic refrigerators (Science Press, 2010).
